# Next-generation biostimulants: molecular insights, digital integration, and regulatory frameworks for sustainable agriculture

**DOI:** 10.3389/fpls.2025.1710899

**Published:** 2025-11-21

**Authors:** Manamele D. Mashabela, Tarekegn Terefe, Pavel Kerchev, Lungile Sitole, Msizi I. Mhlongo

**Affiliations:** 1 Imbewu Metabolomics Research Group, Department of Biochemistry, Faculty of Science, University of Johannesburg, Johannesburg, South Africa; 2 Research Centre for Plant Metabolomics, Faculty of Science, University of, Johannesburg, South Africa; 3 Division of Crop Protection, Agricultural Research Council-Small Grains (ARC-SG), Bethlehem, South Africa; 4 Department of Stress, Development and Signalling in Plants, Estación Experimental del Zaidín, Consejo Superior de Investigaciones Científicas (CSIC), Granada, Spain; 5 Division of Medical Biochemistry and Structural Biology, Department of Integrative Biomedical Sciences, Faculty of Health Sciences, University of Cape Town, Cape Town, South Africa

**Keywords:** agriculture 5.0, biostimulants, omics, climate-smart agriculture, regulation, PGPR, seaweed extracts

## Abstract

The development of biostimulants is undergoing a critical evolution, shifting from empirical applications toward precisely engineered solutions. However, this transition is hampered by fundamental gaps, inclusive of: (1) the absence of temporal-technological frameworks connecting biostimulants development with broader agricultural revolutions, (2) insufficient mechanistic understanding linking molecular modes of action to precision application strategies, and (3) unclear regulatory frameworks and integration pathways for biostimulants within digital agriculture ecosystems (AI/IoT). This review synthesises the evolution of biostimulants through a generational framework (1.0-4.0) and examines their integration with Agriculture 5.0 technologies. We analyse classifications, molecular mechanisms, and regulatory frameworks while evaluating omics-driven precision biostimulant formulations for AI/IoT integration. Our analysis suggests that successful integration requires coordinated molecular validation, regulatory harmonisation, and digital platform development, providing researchers and policymakers with a roadmap for advancing biostimulants science from fragmented research toward systematic, technology-enabled solutions for climate-smart and sustainable agriculture, in line with SDGs 2, 13, and 15.

## 1 Introduction

The pursuit of sustainable agriculture has become an urgent global priority due to the pressing need to meet the demands of a rapidly growing human population, projected to reach nearly 10 billion by 2050. Meeting this demand will require an estimated 70% increase in food production, while reducing agricultural greenhouse gas emissions by 30-40% (IPCC, 2023). This goal is challenged by climate change, the shrinking of arable land, and the intensification of biotic and abiotic stressors (Linnenluecke et al., 2020). Overexploitation and mismanagement of natural resources have further contributed to yield losses and reduced productivity, limiting progress toward Sustainable Development Goals (SDGs) such as Zero Hunger (SDG 2), Climate Action (SDG 13), and Life on Land (SDG 15). For instance, our global land mass comprises 1,400 million hectares (ha) under cultivation, 80% of which is used for crop production. However, this area of arable land continues to shrink at an alarming rate (Raja et al., 2023).

Traditional approaches such as synthetic agrochemicals, genetic engineering, and selective breeding have shown limited success in sustainably addressing these challenges (Mashabela et al., 2023). Although agrochemicals have long supported agricultural productivity, their continued use has contributed to environmental degradation and human health risks, thereby undermining SDGs 3 (Good Health and Well-being) and 14 (Life Below Water). As a result, the agricultural sector is increasingly turning to environmentally sustainable strategies that reduce chemical dependency. Among these, biostimulants have emerged as promising alternatives.

Biostimulants are substances or microorganisms, ranging from natural extracts to biotechnology-derived formulations, that enhance plant growth, improve nutrient uptake, strengthen stress tolerance, and boost overall plant health without harming the ecosystem. These substances stimulate physiological processes in plants, promoting productivity and resilience in an eco-friendly manner. According to Johnson et al. (2023), understanding the biochemical composition, dosage, and application strategies, as well as the modes of action of biostimulants, is essential to support their development and integration into sustainable agricultural practices. This scientific foundation is equally critical for guiding evidence-based policymaking, enabling the formulation of regulatory frameworks that ensure product efficacy, safety, and standardisation across agricultural systems. Robust policy support can accelerate the responsible adoption of biostimulants while aligning national agricultural agendas with global sustainability goals.

Evidence from multiple studies demonstrates the benefits of biostimulants across crops (Dalal et al., 2019; Mansour et al., 2023; Quille et al., 2025). For instance, Lucini et al. (2018) reported that vegetal biopolymer-based biostimulants significantly increased root and leaf biomass in melons while triggering hormonal and metabolic changes that enhanced defence responses. Similarly, Lephatsi et al. (2022) found that a Bacillus-based biostimulant formulation improved morphophysiological traits and induced metabolic reprogramming in maize seedlings. In other crops such as pepper, tomato, okra, and cassava, biostimulants have been shown to improve yield, accelerate ripening, and enhance nutritional quality (Li et al., 2022; Mannino et al., 2020; Canellas et al., 2023).

This review pursues key integrated objectives: to establish a generational classification framework (Biostimulants 1.0-4.0) contextualising technological evolution from empirical extracts to precision engineered formulations; to synthesise molecular mechanisms underlying biostimulants modes of action and examine how synthetic biology (CRISPR, nanotechnology) enables mechanistically informed applications; and to map integration pathways with Agriculture 5.0 technologies including AI-driven discovery, IoT sensors, and adaptive application systems. We further analyse global regulatory landscapes, identifying harmonised strategies to accelerate biostimulants research and adoption. This integrated perspective provides a roadmap for transitioning biostimulants science toward data-driven, digitally integrated solutions supporting climate resilience and sustainable agriculture.

## 2 Biostimulants conceptualised

Understanding emerging scientific endeavours and groundbreaking developments warrants a brief discussion of origin. The science of biostimulants can be traced back to the early 20th century, specifically to the Union of Soviet Socialist Republics (USSR), where Professor Vladimir Petrovich Filatov first conceptualised “biogenic stimulants” in 1933. Filatov theorised that biological materials accumulate substances that stimulate metabolic processes over time (Filatov, 1944; Gordon, 1947; Yakhin et al., 2017; Raja et al., 2023). These materials, through their accumulated metabolites, were believed to provide regenerative properties to organisms, including plants, by suppressing depressive pathological processes resulting from disease or stress. The adoption and refinement of Filatov’s theory gained traction through subsequent contributions. One notable figure was Blagoveshchensky (1945, 1955, 1956), who applied the concept of biogenic stimulants to plant systems. Blagoveshchensky’s pioneering work explored plant adaptation mechanisms and the biochemical nature of stimulatory actions. He defined biogenic stimulants as “organic acids with stimulating effects due to their dibasic properties, capable of enhancing enzymatic activity in plants” (Raja et al., 2023).

As scientific knowledge progressed, the conceptualisation of biostimulants also evolved. Herve (1994) introduced the term “bio-rational products” to describe naturally derived substances with high specificity, low environmental impact, and beneficial effects on non-target organisms. According to Herve (1994), the characterisation of substances as biostimulants should consider their capacity to modulate a plant’s physiological and biochemical activities at low doses and must be ecologically benign, with reproducible beneficial effects on host plants. However, Herve’s framework was broad, encompassing a wide range of substances without a clear delineation of modes of action, which limited mechanistic clarity.

Zhang and Schmidt (1999) refined the concept by providing an evidence-based, metabolic framework for understanding biostimulant function, with an emphasis on empirical analysis, particularly at the molecular and cellular levels. The action of biostimulants was defined from a metabolic perspective, demonstrated by their effects in improving photosynthetic efficiency, enhancing nutrient uptake, and suppressing disease symptoms (Zhang and Schmidt, 1999; Cataldo et al., 2022; Kundu et al., 2022). The elucidation of specific mechanisms, such as hormone modulation, stress mitigation, nutrient uptake and efficiency, as well as the modulation of soil microbial activity, provided a scientifically grounded understanding of the mode of action of biostimulants. These findings laid the foundation for identifying biostimulants as “pre-stress conditioners” or “metabolic enhancers,” a term popularised by James Beard (Schmidt et al., 2003).

The comprehensive review on biostimulants by Kauffman et al. (2007) consolidated knowledge on these substances by proposing an initial classification system based on biostimulant composition and source, inclusive of humic substances (HSs) (Vaughan and Malcolm, 1985; Chen et al., 2004), protein hydrolases (PHs) (Colla et al., 2014), seaweed extracts (SWEs) (Crouch and van Staden, 1993) and beneficial microorganisms (e.g., mycorrhizae and plant-growth-promoting rhizobacteria) (Mosse, 1973; Vessey, 2003), thus providing a coherent framework, linking biostimulants structure to function. Parallel to the systematic classification, groundbreaking mechanistic research conducted on HSs has established these compounds as foundational to biostimulants research. Canellas et al. (2002, 2008) demonstrated that HS function extends beyond nutrition provision, displaying hormone-like activities, which is an initial indicator of physiological signalling by biostimulants, rather than nutrient supplementation. This mechanistic breakthrough was further elucidated by Nardi et al. (2007), who demonstrated through their transcriptomics analysis that HS has the capacity to modulate gene expression patterns associated with nutrient acquisition and stress responses. Together, the Canellas and Nardi research programs transformed understanding of HS from empirical soil amendments to molecularly characterised biostimulants with defined modes of action, establishing methodological frameworks that would influence the broader field of biostimulant science. Basak (2008) further pioneered the systematic symposium and preconditions for biostimulants, considering their origin (natural or synthetic) and effects on plant physiology, as well as the mechanisms of action. His work helped bridge theoretical understanding with practical application and laid the groundwork for integrating biostimulants into sustainable agriculture systems (Basak, 2008; Calvo et al., 2014).

du Jardin’s (2015) work significantly advanced the field of biostimulants by providing a refined definition and comprehensive classification of these substances, which included a systematised categorisation into microbial inoculants, natural extracts, and biochemical compounds. Du Jardin provided mechanistic insights into how biostimulants enhance plant growth and stress resilience, modulating hormones, improving nutrient uptake, and enhancing defence responses. Importantly, he advocated for regulatory frameworks and standardised testing to ensure product consistency and efficacy. This work provided practical guidance for the application of biostimulants in agriculture, emphasising their integration into sustainable practices and the importance of considering both plant and soil health. This holistic perspective and practical focus facilitated a deeper understanding of biostimulants, supporting their effective use in enhancing crop productivity and resilience.

Today, biostimulants research continues to evolve. The field is being shaped by deeper mechanistic studies, the use of -omics technologies, international standardisation efforts, and the integration of biostimulants into smart and climate-resilient farming systems. This historical and conceptual foundation underscores the dynamic progression of biostimulants from anecdotal use to scientifically validated agricultural innovations.

## 3 Classification systems and regulatory definitions

The lack of standardised definitions and terminologies has historically hampered the scientific and regulatory understanding of biostimulants. Overly broad descriptors and vague frameworks have caused confusion in academia and industry, complicating product classification, regulatory enforcement, and global market access (Critchley et al., 2021). Various scientific and governing bodies have attempted to resolve this ambiguity through functional and regulatory definitions. For instance, the European Union (EU) has assigned the term ‘fertilising product’ to biostimulants, whose primary function is to stimulate plant nutrition efficiency, abiotic stress tolerance, crop quality, or nutrient availability, independent of their direct nutrient content (Rouphael and Colla, 2020).

### 3.1 The lack of standardisation and the emergence of harmonised classifications

The divergence between du Jardin’s mechanistic definition and the EU’s regulatory-focused definition has led to challenges in standardisation, resulting in fragmented territorial definitions worldwide (Kumari et al., 2022). These discrepancies lead to ambiguity among stakeholders, inconsistencies in research and development criteria, regulatory complexities resulting from overlapping categories, and market fragmentation due to inconsistent product labelling and claims. Such issues hinder the development of standardised testing protocols and performance benchmarks, impede international harmonisation efforts, and create gaps between scientific research and commercial application. While this review does not seek to resolve these complexities, it echoes the concerns of stakeholders and advocates for a unified, globally accepted definition that combines functional outcomes with mechanistic understanding. Such a framework would facilitate standardised testing, regulatory clarity, and improved market development. Moreover, it would enable the alignment of scientific innovation with policy, ensuring the safe and effective adoption of PBs.

A major step toward this goal was taken by Yakhin et al. (2017), who proposed a comprehensive definition of PBs as “formulated products of biological origin that improve plant productivity as a consequence of the novel, or emergent properties of the complex of constituents, and not as a sole consequence of the presence of known essential plant nutrients, plant growth regulators, or plant protective compounds.” This definition, which builds on du Jardin’s work, emphasises the importance of emergent functionality over constituent identity. Additional contributions include Bulgari et al. (2015), who suggested including physiological responses and mechanisms of action in PB definitions, while Basak (2008) stressed the importance of origin and functionality. Over time, definitions have expanded to include microbial products, microbial metabolites, and mixtures thereof, whether they contain viable organisms, provided they result in measurable benefits for plants (Rouphael and Colla, 2020).

Nevertheless, classification remains a challenge due to the diversity of PB sources (bacteria, fungi, algae, higher plants), processing methods, and complex modes of action. Intellectual property (IP) rights, patents, and proprietary formulations further complicate categorisation by introducing novel combinations or functionalities that fall outside traditional regulatory boxes. IP-protected products often contain unique metabolites or synergistic blends that challenge rigid regulatory structures. These innovations may prompt the creation of new subcategories, but they also highlight the urgency for adaptable classification systems. For example, some microbial products function through indirect pathways, such as quorum sensing or the release of secondary metabolites, while others act more directly via nutrient solubilisation or phytohormone modulation. Additionally, definitions continue to evolve, and given that the target application is commercial, the industry will play a key role in defining and promoting the concept of biostimulants.

Efforts to classify PBs date back to Filatov’s 1951 work, and, like their definitions, these systems have undergone considerable evolution. Some definitions and classifications were more divergent and less refined prior to the consolidation of categories based on multiple classifiers. Early attempts lacked scientific rigour and often grouped PBs with fertilisers or plant growth regulators (du Jardin, 2015). The emphasis was on non-nutrient and non-hormonal functions, such as improved nutrient uptake, stress mitigation, or stimulation of plant metabolism, without directly supplying nutrients or acting as hormones (Calvo et al., 2014). As such, du Jardin (2015), from an exhaustive literature search through 250 articles, proposed 8 categories of biostimulants inclusive of 1) humic substances, 2) complex organic materials, 3) beneficial chemical elements, 4) inorganic salts, 5) seaweed extracts, 6) chitin and chitosan derivatives, 7) anti-transpirants and 8) free amino acids and other N-containing substances, with a notable exclusion of microorganisms due to their then classification as biopesticides and sources of plant hormones (biofertilizers) due to certain regulatory frameworks (Vessey, 2003; Sible et al., 2021). However, as research advanced, it became clear that microbial agents, such as plant growth-promoting rhizobacteria (PGPR) and mycorrhizal fungi (AMF), play crucial roles in plant growth, stress resilience, and nutrient efficiency, functions consistent with PBs (Vessey, 2003; Rouphael and Colla, 2020).

This overlap blurred the lines between microbial inoculants and chemical biostimulants. As a result, researchers such as du Jardin and Calvo et al. (2014) have advocated for an expanded definition that includes both microbial and non-microbial PBs, based on shared mechanisms of action. This shift in understanding has led to the formal recognition of two broad categories of PBs: microbial PBs, such as PGPR and AMF, and non-microbial PBs, including seaweed extracts, humic acids, and protein hydrolysates. As the field continues to expand, classification systems will need to remain dynamic to accommodate new biotechnologies, hybrid products, and multi-functional formulations. Future definitions should integrate compositional identity, functional outcomes, and regulatory requirements, while remaining flexible enough to include innovation.

### 3.2 Toward a generational classification framework

**Figure 1:**
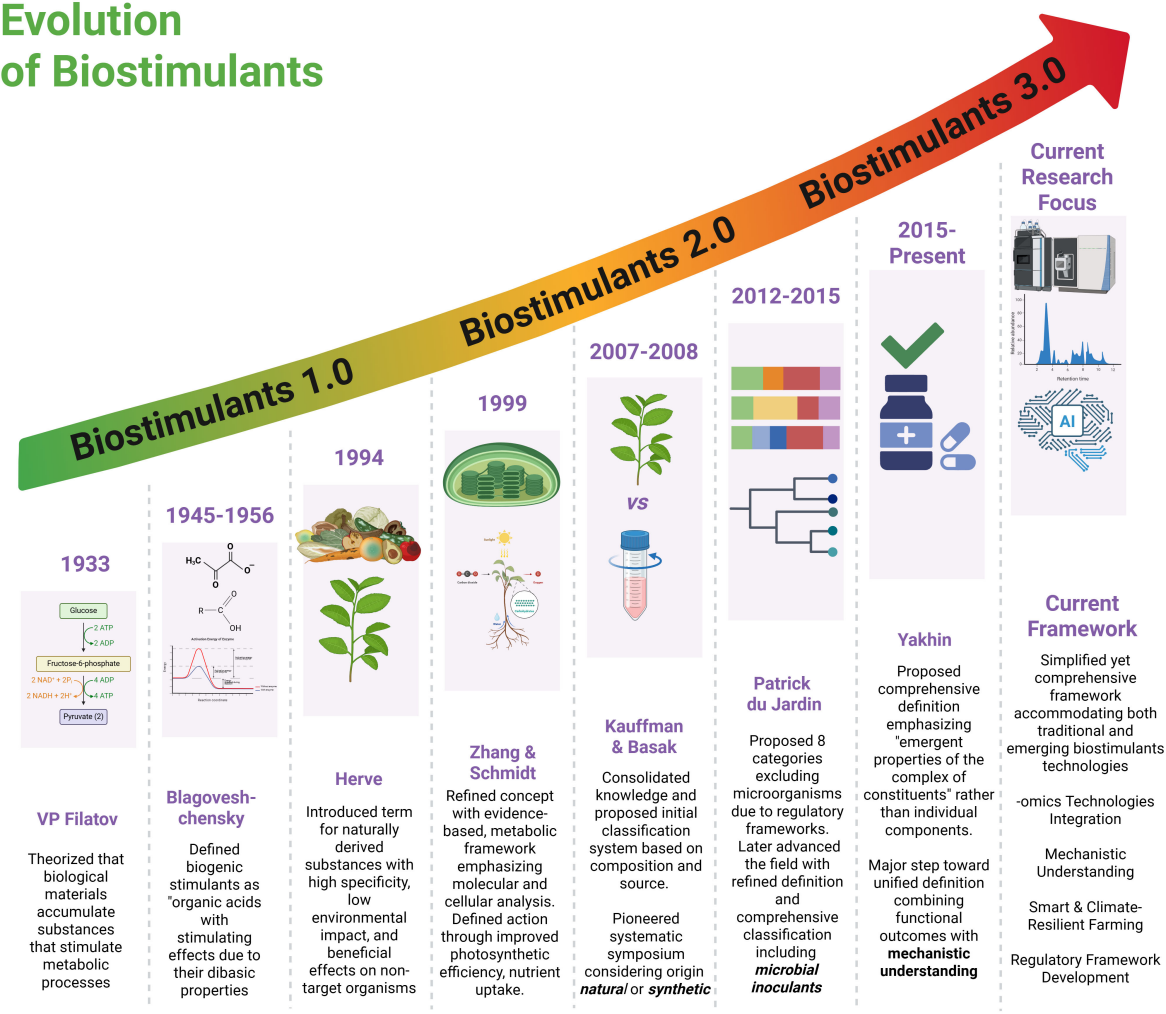
Evolution of biostimulants from 1.0 to 3.0 across nine decades of development (1933-present). The timeline progresses from early theoretical foundations through systematic classification periods to current AI-integrated approaches (2015-present). Each generation represents increasing sophistication: Biostimulants 1.0 focused on basic biological understanding, 2.0 emphasised evidence-based metabolic frameworks and regulatory development, while 3.0 integrates omics technologies, mechanistic understanding, and smart climate-resilient farming with comprehensive regulatory frameworks.

The challenges in biostimulants classification outlined above reflect not only compositional diversity but also the rapid technological evolution of the field. While existing classification systems focus primarily on source materials and compositional identity, they may not fully capture the temporal-technological progression that characterises biostimulants development. To stimulate discussion and guide future research, we present a preliminary conceptual framework that tentatively organises biostimulants according to apparent technological sophistication and underlying scientific paradigms (**Figure 1**). This generational nomenclature draws inspiration from established approaches in related fields, such as the industrial revolution (IR 1.0 - 4.0) and agriculture (1.0 - 5.0), which have successfully provided temporal-technological frameworks for understanding sectoral evolution (Fountas et al., 2019; Haloui et al., 2024). Most importantly, unlike compositional classifications, which answer “what,” biostimulants address “when” and “how” different technological approaches emerged and evolved to advance biostimulants research and application. This exploratory framework requires rigorous validation through comprehensive data collection, industry surveys, and longitudinal analysis before it can be considered a robust classification system.

The earliest generation represents traditional, empirically derived natural extracts and products, characterised by a limited mechanistic understanding. These products, including crude extracts of seaweeds, compost material and fermented organic matter, were applied based on observational benefits rather than scientific validation. We have termed this generation, Biostimulants 1.0 (pre-2000), defined by minimal characterisation, largely unknown modes and mechanisms of action. Biostimulants 2.0 (2000-2015) was defined by advances in microbiology and physiology, which enabled mechanistic insights into the action of biostimulants, leading to the development of standardised microbial inoculants, including plant growth-promoting rhizobacteria (PGPR) and arbuscular mycorrhizal fungi (AMF), alongside refined natural extracts with identified active compounds. This phase also saw quality control and efficacy testing.

The current generation, Biostimulants 3.0 (2015–present), can be defined by the integration of multi-omics technologies, systems biology, and biotechnology to create precision bioformulations tailored to specific genotypes and environments (Ma et al., 2022; Wang et al., 2025). Leveraging insights from omics technology, alongside synthetic biology and nanotechnology, these products feature enhanced delivery systems and predictable outcomes from targeted applications (Tyagi et al., 2023; Chen et al., 2024). Looking forward, Biostimulants 4.0, emerging post-2020, would represent the convergence of omics-informed biostimulants development with Fourth Industrial Revolution (4IR) technologies, characterised by AI-driven discovery platforms, IoT-enabled sensor systems, and real-time monitoring and adaptive applications strategies (Mansoor et al., 2025; Fountas et al., 2024). These smart biostimulant systems would employ predictive algorithms to optimise timing and dosage, while novel delivery mechanisms respond dynamically to environmental cues, exemplifying the shift towards AI-integrated, IoT-responsive agriculture (Wang et al., 2025; Miller et al., 2025).

This generational roadmap illustrates not only the scientific and technological maturation of biostimulants but also the shifting paradigms that underpin their development and application. Each successive phase reflects a progression from empirical observation to mechanistic understanding, from standardised formulations to precision bioengineering, and now toward digitally integrated smart systems. However, it is worth noting that generational boundaries are not absolute; technological transitions occur gradually, and hybrid approaches often combine elements from multiple generations. While Biostimulants 3.0 remain the prevailing paradigm in both research and commercial deployment, the conceptual emergence of Biostimulants 4.0 signals a transformative trajectory aligned with the broader vision of Agriculture 5.0. To appreciate this transition, it is necessary to examine the technological and biotechnological innovations that are redefining precision, targeted action, and the design of next-generation bioformulations. While a comprehensive analysis of this nomenclature warrants dedicated review, this preliminary framework offers a complementary lens through which to understand the evolution of biostimulants. Rather than replacing compositional classifications, it provides a temporal technological context that may prove valuable for researchers, industry stakeholders, and policymakers navigating the rapidly evolving biostimulants landscape. The framework also highlights the trajectory toward increasingly sophisticated, data-driven bioformulations that integrate seamlessly with digital agriculture platforms, a progression that aligns with the broader transformation of agricultural systems toward precision, sustainability, and technological integration.

While classification frameworks establish what constitutes a biostimulant and how these categories have evolved, the next challenge lies in understanding how biotechnology is reshaping their development. Advances in microbial engineering, synthetic biology, and systems biology provide the foundation for precision biostimulants, moving beyond compositional identity toward tailored, mechanism-driven solutions.

## 4 Molecular engineering and biostimulants 3.0: the 3^rd^ generation

**Figure 2:**
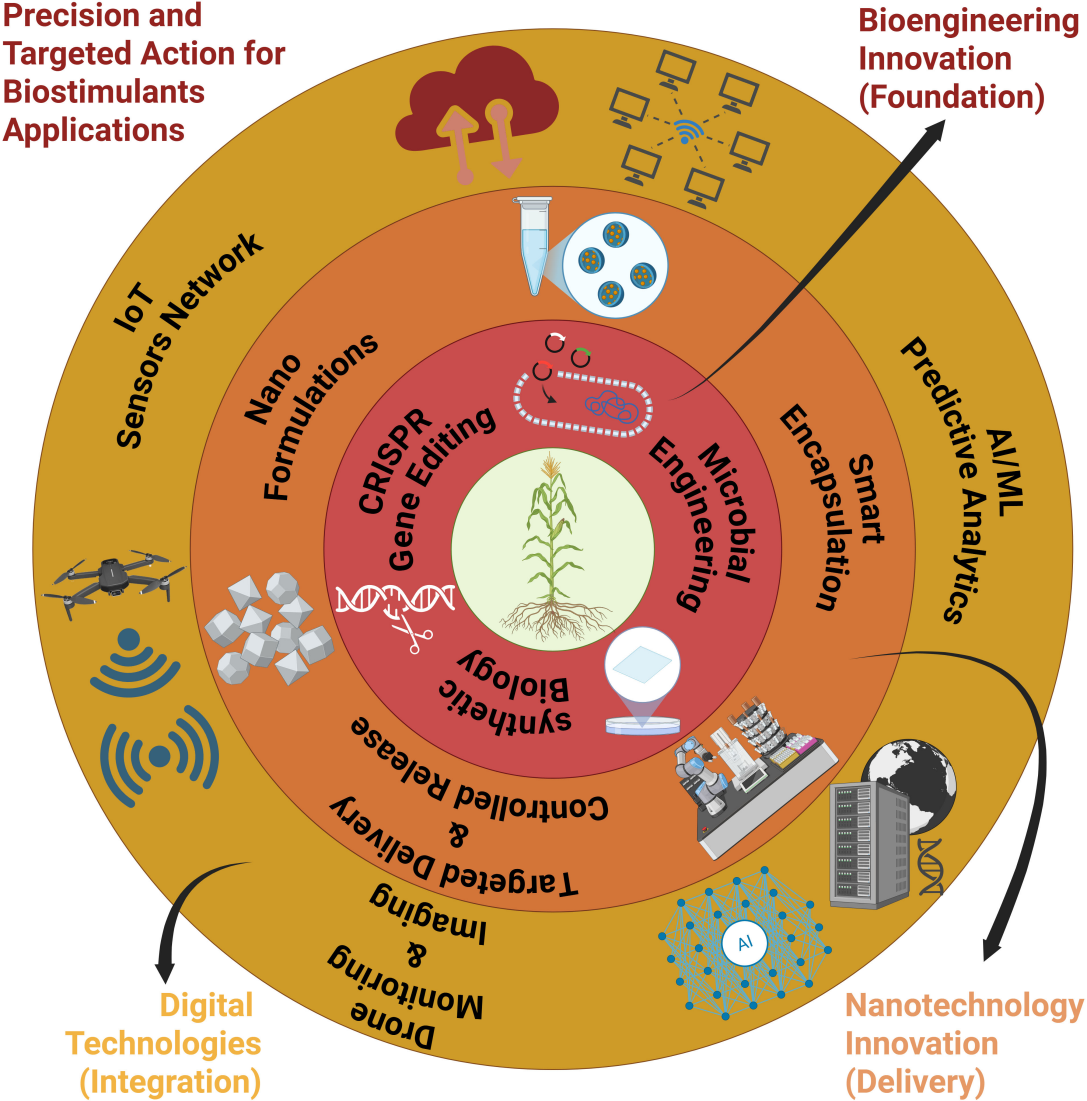
Concentric framework for precision biostimulants applications integrating bioengineering innovation across multiple technological layers. The innermost core represents synthetic biology and CRISPR gene editing for microbial engineering. Progressive outer rings encompass nano-formulations and encapsulation technologies, smart delivery systems with predictive AI/ML analytics, IoT sensor networks, and digital technologies for monitoring and control. The outermost layer focuses on precision application through the use of drone technology and innovative nanotechnology. This integrated approach enables the targeted and controlled release of biostimulants, enhancing agricultural efficiency.

The advancement of biostimulants has shifted from empirical applications toward precise, knowledge-driven and biotechnology-driven innovations. The 3^rd^ generation (Biostimulants 3.0) of biostimulants, as outlined above, moves beyond traditional plant growth and defence enhancement, aiming to deliver more targeted, advanced, and sustainable solutions for agriculture (Nephali et al., 2022). This concept represents the evolution of biostimulants in terms of formulation, application, and mechanisms, incorporating new scientific insights, technologies, and approaches. Synthetic and engineered biostimulants represent a cutting-edge approach, utilising synthetic biology and nanotechnology to create novel compounds and microorganisms to enhance functionality, specificity, and consistency of performance (Jorquera et al., 2016; Wei et al., 2022) (**Figure 2**). These emerging categories reflect the evolving understanding of biostimulants and their diverse mechanisms of action. This generation potential creates opportunities for sustainable agriculture (Nephali et al., 2020; Garg et al., 2024). This mirrors the transition from Agriculture 1.0, which is characterised by traditional, low-input farming, to Agriculture 3.0, characterised by precision, sustainability, and biotechnological integration.

### 4.1 Precision and targeted action

With increasing demand for sustainable and efficient agricultural inputs, the development of biostimulants has shifted toward precision and targeted mechanisms. This evolution builds upon early work on waste-derived and natural-extract biostimulants, which highlighted the value of circular bioeconomy practices (Rouphael and Colla, 2020). This approach urged producers to valorise their by-products by using agricultural products with improved environmental profiles (Khan et al., 2020; Rodrigues et al., 2022). Interestingly, valorised agricultural by-products, such as potato skins, are enriched in diverse secondary metabolites due to their exposure to environmental stressors. For instance, potato peel is enriched in steroidal alkaloids, which are associated with defence against bacterial, fungal, and insect pathogens (Fritsch et al., 2017; Xu and Geelen, 2018).

To this end, researchers have strategically explored refined bioactive compounds and secondary metabolites, peptides, or signalling molecules and microbe-derived metabolites, for precision and targeted application by using well-characterised metabolites to achieve specific and predictable responses in crops, aligning bioactive compounds with known molecular pathways (Aremu et al., 2015; Shukla et al., 2019). In their study, Aremu et al. (2015) reported secondary metabolites as potent stimulators of root and shoot development. The application of secondary metabolites on *Eucomis autumnalis* revealed enhanced development of underground parts following treatment with seaweed (*Ecklonia maxima*)-derived phenolic compounds (Aremu et al., 2015).

An earlier study investigating the effects of seaweed extracts on plant abiotic stress tolerance revealed ANE-influenced gene expression in *A. thaliana* under salt stress (Jithesh et al., 2012). Transcriptomic analysis showed that PME expression was induced under moderate salt stress but decreased at higher salt concentrations. Anti-coexpression analysis further revealed overrepresented genes involved in ion transport, particularly ATPase-mediated transmembrane transport, suggesting that the extracts modulate key processes related to sodium accumulation and stress tolerance. These findings were the earliest to highlight the molecular mechanisms by which bioactive compounds and natural extracts confer salt stress tolerance, providing insights into their potential applications in improving plant resilience to abiotic stresses (Jithesh et al., 2012). This specificity has led to the refinement of bioactive compounds, peptides, microbial metabolites, and signalling molecules to interact with known molecular pathways. Beyond discovery, advanced purification techniques, including chromatography, membrane separation, and metabolomic profiling, can be utilised to isolate key bioactive components, ensuring specificity in their applications (Battacharyya et al., 2015). Combined with transcriptomics and metabolomics, these insights allow developers to link compound application with gene expression changes, offering predictable outcomes and potential for regulatory approval. Studies using GC-MS and qNMR have distinguished seasonal and manufacturer differences in seaweed extract compositions (Craigie et al., 2008; Craigie, 2011). Meanwhile, bioassay-guided fractionation enables the identification of bio-efficacy-driving fractions. Innovations like encapsulation and co-formulation with stabilising agents improve shelf life and bioavailability, while biopolymer integration (e.g., chitosan, starch, alginate) enhances delivery and environmental compatibility (El-Tarabily et al., 2020; Khan et al., 2020).

The rise of precision biostimulants, those aligned with defined biochemical targets and regulatory tolerances, has profound policy implications. As governments and international bodies push for data-driven, sustainable agriculture, precision PBs offer a scalable solution to reduce reliance on synthetic inputs, improve crop quality, and ensure compliance with evolving agricultural legislation.

### 4.2 Biotechnological innovations, microbial inoculants and bioengineering

Recent advances in microbial engineering, nanotechnology, and synthetic biology have enabled the development of the third generation of biostimulants, which are more precise, resilient, and multifunctional. These biotechnological innovations aim to overcome traditional limitations, such as inconsistent field performance and low stability, while delivering tailored responses to specific environmental or physiological conditions.

Synthetic biology allows for the design of microbial strains with tailored biosynthetic capabilities. For example, bacterial strains can be engineered to overproduce phytohormones (e.g., indole-3-acetic acid), siderophores, or enzymes involved in phosphate solubilisation (Orozco-Mosqueda et al., 2023). CRISPR-Cas9 and related tools enable precise genetic modifications that enhance microbial resilience and specificity, ensuring better colonisation of plant roots and more predictable outcomes (Albright et al., 2022; Chen et al., 2024). Additionally, emerging RNA-based and gene-editing technologies offer new avenues for developing biostimulant-responsive crops. CRISPR-Cas9 has enabled targeted modifications in genes associated with hormone signalling, stress responses, and nutrient transport. Meanwhile, RNA interference (RNAi) approaches modulate specific stress-related pathways without altering DNA, making them promising tools for fine-tuned crop management (Gao, 2021; Zhao et al., 2024; Gogoi et al., 2025).Metabolite optimisation can also be achieved through pathway refactoring, which involves restructuring native biosynthetic routes to improve flux toward desired metabolites while reducing byproducts (Gharat et al., 2025). This enables higher yields of key compounds, such as polyamines, amino acids, or osmolytes, which contribute to plant stress tolerance and metabolic enhancement.

An example is the engineering of *Pseudomonas fluorescens* strains to enhance drought tolerance in wheat by increasing ACC deaminase activity and promoting the production of osmoprotectants. Similarly, *Bacillus subtilis* has been modified to enhance lipopeptide biosynthesis, increasing antifungal activity and root adhesion properties (Marrone, 2021; Albright et al., 2022). In other studies, *Pseudomonas putida* and *Rhizobium meliloti* have been modified to express chitinase genes, enhancing biocontrol against fungal pathogens (Bagwan, 2010; Ali et al., 2021). Similarly, *Pseudomonas protegens* Pf-5 has been engineered to fix nitrogen by utilising nitrogenase genes from P. stutzeri, thereby improving growth in nitrogen-deficient soils (Setten et al., 2013). These engineered strains represent a significant step toward reliable and application-specific microbial inoculants for diverse agroecosystems. Although biotechnology offers the molecular tools to design novel bioformulations, their field performance often depends on how effectively they can be delivered to plants. Here, nanotechnology provides the critical link, offering smart carriers and encapsulation systems that ensure stability, controlled release, and responsiveness to environmental cues.

Nanotechnology offers a transformative approach to biostimulants delivery through nanosystems, which are integrated nanoscale platforms that combine engineered nanocarriers, encapsulated bioactive compounds and responsive release mechanisms, enabling the formulation of nanobiostimulants and nanofertilizers that are more stable, efficient, and environmentally responsive. These nanoformulations enhance absorption, boost photosynthesis, improve stress tolerance, and facilitate precise nutrient release and delivery under controlled environmental conditions through precisely size-controlled and surface-modified carriers (Tyagi et al., 2023; Ayenew et al., 2025) (**Figure 2**). For example, chitosan nanoparticles loaded with stress-alleviating agents have demonstrated improved translocation and mitigation of salt stress in maize (Oliveira et al., 2016). Studies have revealed that nanocarriers, such as ZnO or SiO2 composites, improve bioavailability, reduce nutrient losses, and increase crop yields. In addition to targeted nutrient delivery and nutrient efficiency, NFs also contribute to the prevention of water resource and atmospheric contamination (Dwivedi et al., 2016; Ayenew et al., 2025). The concept of NF technology is highly innovative, utilising physical, chemical, and biological methods for formulation.

Production methods vary, including top-down (e.g., grinding and milling) and bottom-up (e.g., co-precipitation, biosynthesis) approaches. Of particular interest is the green synthesis of nanoparticles using microbes, fungi, and plant extracts, techniques that avoid the use of toxic chemicals and align with sustainability goals (Jahangirian et al., 2020; Das Mahapatra et al., 2022). For example, bacterial and fungal strains capable of reducing metal ions offer an environmentally friendly means of fabricating bioactive nanomaterials. Though still largely experimental, these approaches have the potential to complement biostimulant treatments by enhancing compatibility and response efficiency. Despite their promise, nanobiostimulants raise biosafety concerns, and their environmental fate and toxicity require further assessment. Regulatory frameworks should evolve to establish acceptable use thresholds, define nano-specific labelling, and promote responsible innovation. Their integration into policy will be essential to support precision agriculture while safeguarding ecosystems.

### 4.3 Mechanistic basis for technology integration

While the preceding discussions outlined biotechnological innovations in biostimulants development, understanding how these technologies function at a molecular and cellular level is paramount for rational product design and predictive application strategies. This systems-level understanding of mechanistic foundations applies to nanotechnology-enhanced delivery, gene editing-mediated responsiveness, and synthetic biology-programmed microbial functions.

#### 4.3.1 Nanotechnology: cellular and molecular mechanisms

The superior performance of nanocarriers derives from specific cellular and molecular mechanisms: (1) enhanced cellular uptake, (2) controlled release kinetics, and (3) targeted delivery (Oliveira et al., 2016). Cellular uptake is facilitated through size-dependent penetration of cell wall pores (5–20 nm) and endocytosis-like membrane internalisation. Recent studies have demonstrated that chitosan nanoparticles, for instance, achieve 10-to 12-fold faster cellular penetration and uptake compared to bulk formulations (Massawe et al., 2025; Narkhede and Bhamare, 2025). On the other hand, controlled release kinetics have been shown to enable sustained bioactive compound availability. Sampedro-Guerrero et al. (2023) reported that alginate-encapsulated phytohormones exhibited biphasic release characterised by a burst phase (30-40% release) over 0–6 hours following application, and a subsequent sustained phytohormone release over 6–120 hours, while maintaining optimal concentration (10^-8^–10–^6^ M). This display contrasts with an 80% degradation of bulk phytohormone application within 12 hours of administration. While targeted cellular and subcellular delivery directs functional nanocarriers to specific organelles. According to Kumari et al. (2024), conjugated nanocarriers can achieve up to 10-15-fold higher local (target cell) concentrations. For instance, in their study, Răcuciu et al. (2022) demonstrated that transit magnetite nanoparticles can deliver magnetite to chloroplasts, inducing oxidative stress that activates the plant’s defence/antioxidant mechanisms to protect the photosynthetic machinery of maize plants. Moreover, rhizosphere applications of nanoparticles are known to modify soil chemistry and microbiology while increasing nutrient absorption. The study by Muhammad et al. (2022) demonstrated effective drought mitigation, along with improved wheat plant water relations, chlorophyll, proline, phenolics, and grain quality, as well as yield and their associated traits, compared to the stressed treatments.

#### 4.3.2 CRISPR and synthetic biology: engineering biostimulants’ responsiveness

Gene editing technologies enable the creation of crop varieties with enhanced biostimulant sensitivity through targeted genetic modifications. Examples include receptor engineering, which involves introducing point mutations that increase hormone binding affinity. For instance, TIR1-F79A modifications enhance IAA binding, enabling plants to respond to lower biostimulant-derived auxin concentrations with equivalent root proliferation (Yamada et al., 2018; Gao et al., 2024). CRISPR-mediated promoter modification has also been shown to increase constitutive expression of high-affinity phosphate (PHT1 family) and nitrate (NRT2.1) transporters for higher nutrient accumulation and improved nutrient-use efficiency (Singh et al., 2025). At the microbial level, synthetic biology enables the construction of intelligent microbial biostimulants and microbial consortia with environmentally responsive genetic circuits. For instance, stress-sensing circuits can be combined with effector modules expressing stress-responsive pathways (ACC deaminase) to achieve superior performance of microbial biostimulants. As an example, a 15-member synthetic microbial community (SynCom) from the *Brachypodium distachyon* rhizosphere was developed using network and cultivation-based methods. Genomic and phenotypic analyses revealed multiple plant growth-promoting traits, including the synthesis of osmoprotectants and ion transport. The SynCom remained stable, enhanced drought resilience, and preferentially colonised root tips under stress, demonstrating its potential as a scalable tool for studying and improving plant–microbe interactions (Yadav et al., 2025). Additionally, phyllosphere-modulating synthetic communities (PMS) applied to pakchoi were shown to increase biomass by 40-70% and chlorophyll content by ~15% through the secretion of phytohormones and siderophores (He et al., 2024), while nutrient-responsive circuits employ PhoB/PhOR sensors that activate phosphate-solubilising functions in microbes when phosphate concentrations fall below a certain threshold, thereby increasing and sustaining plant-available phosphorus (Timofeeva et al., 2022). These mechanistic insights provide rational foundations for the design of precision biostimulants, predictive application strategies, and integration with digital agriculture platforms.

Taken together, these innovations illustrate the accelerating trajectory of biostimulants research from empirical applications to precision bioengineering and the emerging integration with digital agriculture. However, the pace of technological progress has outstripped the development of clear regulatory frameworks. Whereas Biostimulants 1.0 and 2.0 operated within relatively simple product categories, the advanced formulations of Biostimulants 3.0 and the emerging concepts of 4.0 challenge existing definitions, approval systems, and safety standards. Novel categories such as nanobiostimulants, genetically engineered inoculants, and AI-informed application systems do not yet fit neatly into current legislation, creating uncertainty for producers, policymakers, and end-users alike. This underscores the urgent need for coherent, science-driven regulatory frameworks that can keep pace with innovation while ensuring product safety, efficacy, and global market accessibility.

## 5 Global regulatory landscape and harmonisation pathways

​The regulatory challenges created by advanced biostimulant technologies reflect broader fragmentation in global biostimulant governance. Inconsistent definitions, divergent registration procedures, and variable data requirements across jurisdictions have created a complex web of region-specific policies that slow product development, inflate compliance costs, and limit market access, particularly for small and medium-sized enterprises (SMEs) that dominate the sector (La Torre et al., 2016; Caradonia et al., 2019). The European Union (EU) has led efforts to address these challenges through coherent, science-based frameworks that strike a balance between innovation and safety assurance.

### 5.1 Regulatory developments in the European Union and beyond

**Figure 3:**
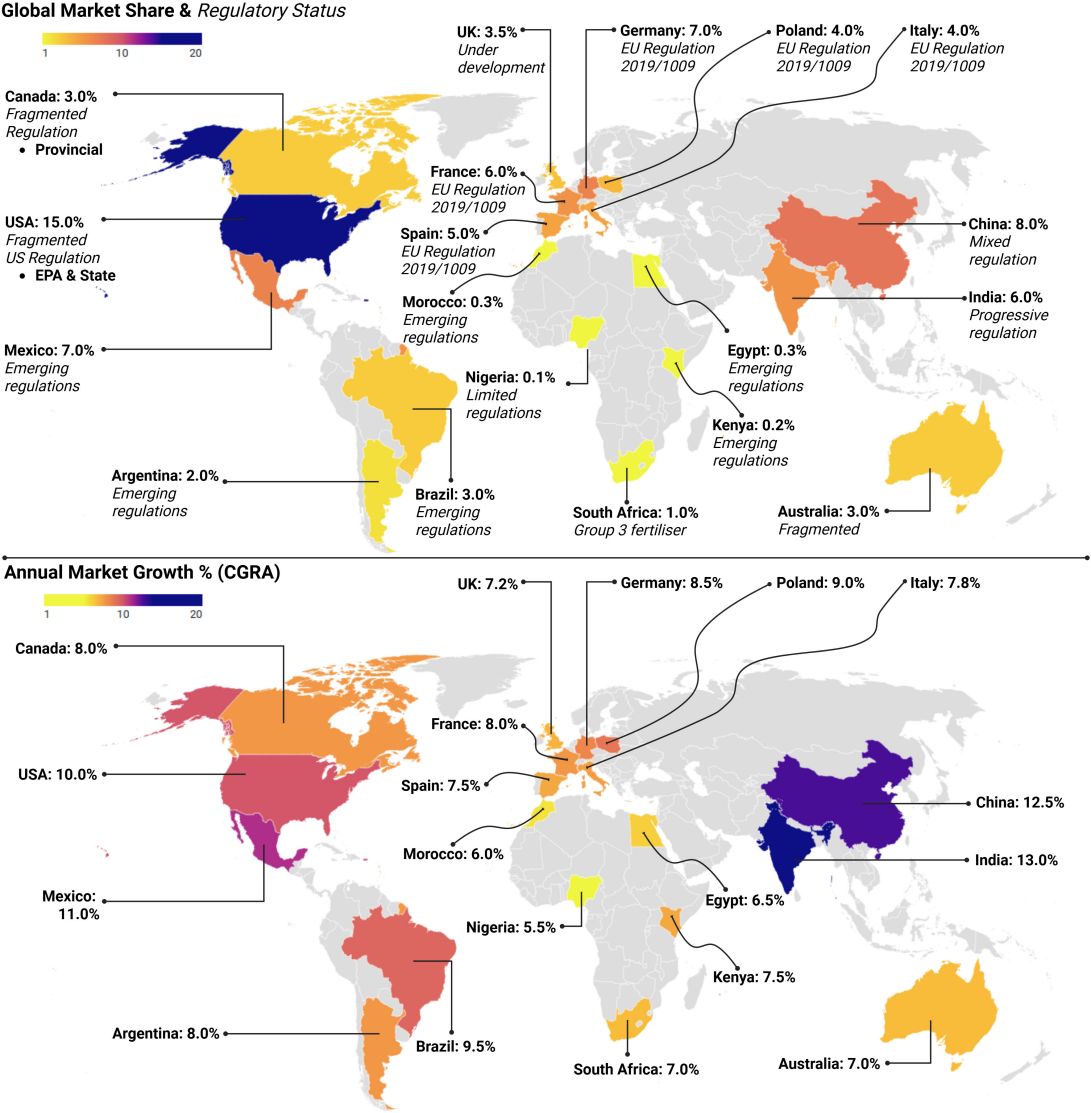
Global biostimulants market analysis showing market share distribution and annual growth rates by country with regulatory status indicators. The upper map displays current market shares, with the USA leading at 15.0%, followed by China (8.0%) and Mexico (7.0%). Regulatory frameworks vary from fragmented (e.g., the USA, Canada, Australia) to emerging (e.g., Brazil, Argentina, African nations) to established EU regulations. The lower map displays annual market growth rates (CAGR), with India and China exhibiting the highest growth rates (13.0% and 12.5%, respectively), while established markets demonstrate moderate growth. Colour coding indicates growth intensity from low (yellow) to high (purple).

The lack of uniformity in biostimulants regulation has created a complex web of region-specific policies, which often differ in definitions, registration procedures, and data requirements (Yakhin et al., 2017). The European Union (EU) has been at the forefront of establishing clear regulations for PBs. Regulation (EU) 2019/1009, which came into force in July 2022, marked a critical shift by officially recognising PBs as a distinct category under EU fertilising products. These are now defined as substances that stimulate nutrient-use efficiency, tolerance to abiotic stress, crop quality, or nutrient availability in soil or the rhizosphere, irrespective of nutrient content (Rouphael and Colla, 2020; Caradonia et al., 2019). This replaced earlier regulatory ambiguity under Regulation (EC) No. 2003/2003 (which lumped PBs under fertilisers) and Regulation (EC) No. 1107/2009 (covering plant protection products). Such misclassification hindered PB innovation and investment, especially from SMEs, due to the stringent and ill-fitting regulatory demands (La Torre et al., 2016). Regulation (EU) 2019/1009 introduced clear standards for product efficacy, safety, and labelling, as well as a harmonised registration pathway that includes microbial and non-microbial PBs (Meena et al., 2025). All EU member states must adhere to this unified system for products bearing the CE mark (**Figure 3**), although national systems can still operate in parallel during a transitional phase. The harmonised EU definition has already improved trade predictability and product confidence across the bloc.

In contrast, the United States still lacks a federal definition for biostimulants. Oversight is divided: the Environmental Protection Agency (EPA) regulates claims related to plant protection, while state-level departments of agriculture handle plant growth claims, each with different registration requirements, fees, and definitions (du Jardin, 2015; Caradonia et al., 2019). This regulatory fragmentation has created substantial barriers for companies, particularly those seeking multi-state approval. A proposed Plant Biostimulants Act aims to define PBs federally and streamline regulation, but it remains pending as of 2025 (Meena et al., 2025). India has adopted a more progressive approach, incorporating PBs under the Fertiliser Control Order through the 2021 and 2024 amendments. PBs are formally recognised and regulated as biofertilisers with defined efficacy and safety testing requirements, enabling national consistency and commercial clarity.

Across Africa, the regulatory landscape for PBs remains highly variable. South Africa is a notable exception, classifying PBs as “Group 3 Fertilisers” under its Regulations Regarding Fertilisers N.R. 732 of 10 September 2012. The definition includes seaweed extracts, PGPR, organic acids, and their combinations (Caradonia et al., 2019). However, in many other African nations, regulatory clarity is still lacking. Countries like Morocco, Egypt, and Kenya have begun requiring efficacy trials, safety assessments, and technical dossiers for biostimulant registration; however, they lack harmonised definitions or clear regulatory categories. Other countries, including Nigeria, Ghana, Rwanda, and Uganda, face challenges related to infrastructure, awareness, funding, and technical capacity (Sayyed et al., 2014; Raimi et al., 2021). In these contexts, the absence of formal frameworks limits local innovation and deters foreign investment. The Asia-Pacific region faces similar inconsistencies. For instance, China lacks a specific category for PBs, often grouping them under microbial or water-soluble fertilisers. Registration must be obtained through the Ministry of Agriculture and Rural Affairs, but the lack of a unified biostimulants framework introduces regulatory ambiguity and impedes product development (Fan, 2017).

This fragmentation not only stifles innovation but also inflates compliance costs and delays market entry, especially for smaller firms. As PBs become central to climate-resilient agriculture, harmonisation across emerging markets will be critical to ensuring equitable access and effective implementation. Evidently, these regional disparities underscore the need for global harmonisation. Current efforts toward this goal include international forums and trade associations advocating for common definitions and guidelines. However, progress has been slow. Therefore, it is recommended that the global biostimulants community strive towards the establishment of an international regulatory advisory body for biostimulants under the sponsorship of the Food and Agriculture Organisation (FAO) or the World Health Organisation (WHO) (Raimi et al., 2021; Meena et al., 2025), the development of a centralised global database for approved substances, and mutual recognition and agreements between countries with comparable safety standards. Furthermore, increased funding for regulatory research and stakeholder engagement across regions will be crucial to building trust and fostering alignment (Meena et al., 2025).

Over time, it would be quite useful to create synergies in European regulations- as the leading party in biostimulants development- and legislation on PBs outside the EU to maintain the same quality standards and avoid creating confusion during international exchanges (for example, the use in Brazil and India of the name “Biofertilizers” to identify these substances could generate confusion among states). Harmonised rules could facilitate the establishment of a robust risk assessment framework (du Jardin, 2015), allowing for the safe circulation of these new products in global markets and legitimising this category of substances. The European Regulations represent legislative frameworks affecting European countries and those countries trading with one of the world’s leading economic powers. For these reasons, the European legislative framework often serves as a model and is imposed in other regions outside Europe. This could lead to a unified global adoption of a harmonised regulatory framework.

### 5.2 Aligning regulation with science and innovation

Effective regulatory harmonisation requires integrating current scientific methodologies into assessment frameworks. A crucial step toward alignment involves incorporating scientific methods into regulatory assessment frameworks. Science-based risk assessments utilising systems biology approaches and omics technologies (transcriptomics, metabolomics, proteomics) can help clarify the mode of action, safety, and efficacy of biostimulants (Baghdadi et al., 2022). Tiered testing protocols could be particularly useful, enabling differential regulation based on product complexity and potential environmental impact. Such a framework would support both innovation and safety without imposing undue burdens on producers, particularly SMEs, which form the backbone of the biostimulants industry and are disproportionately impacted by regulatory inconsistencies (Caradonia et al., 2019).

Additionally, international trade agreements and mutual recognition treaties can reduce market fragmentation and redundant registration processes. By incorporating mutual recognition of product approvals based on shared scientific standards into bilateral and regional trade pacts, countries can enhance regulatory transparency and reduce time-to-market for new biostimulant products (Backer et al., 2018). However, such alignment must also include capacity building in emerging economies, particularly in sub-Saharan Africa and the Asia-Pacific region, where regulatory infrastructures may be underdeveloped. Investments in national laboratories, regulatory bodies, and standardised field trial systems are essential to foster reliable assessments and enable participation in global markets (Meena et al., 2025). Public-private partnerships (PPPs) represent another strategic avenue to facilitate the translation of science into policy. Collaborative efforts among governments, research institutions, and industry stakeholders can lead to the development of co-created regulatory guidelines, real-time data sharing platforms, and pre-competitive research consortia that provide evidence for informed policymaking (Backer et al., 2018).

Simultaneously, digitalisation of regulatory systems can improve efficiency and traceability. A centralised regulatory harmonisation portal could streamline submissions, approvals, and compliance checks across regions. Finally, globally recognised sustainability labelling and certification schemes could standardise product quality and claims, enhance consumer trust, and guide regulators. These schemes, akin to organic certification systems, would also incentivise the adoption of eco-friendly innovations. Supporting SMEs through reduced regulatory fees, technical assistance, and simplified compliance pathways could further democratise access to global markets (Calvo et al., 2014; Caradonia et al., 2019). Ultimately, aligning regulatory frameworks with science and commercial integration requires coordinated, inclusive, and evidence-driven strategies that account for the diversity of global agricultural systems and stakeholders.

Overall, while strides have been made in some regions, notably India, the global regulation of biostimulants remains inconsistent and fragmented. This presents significant obstacles to market expansion, innovation, and sustainable agricultural development. Coordinated international action is needed to develop harmonised frameworks that support scientific progress, ensure product efficacy and safety, and promote equitable access across diverse agricultural systems.

## 6 Integration with digital agriculture: towards biostimulants 4:0

**Figure 4:**
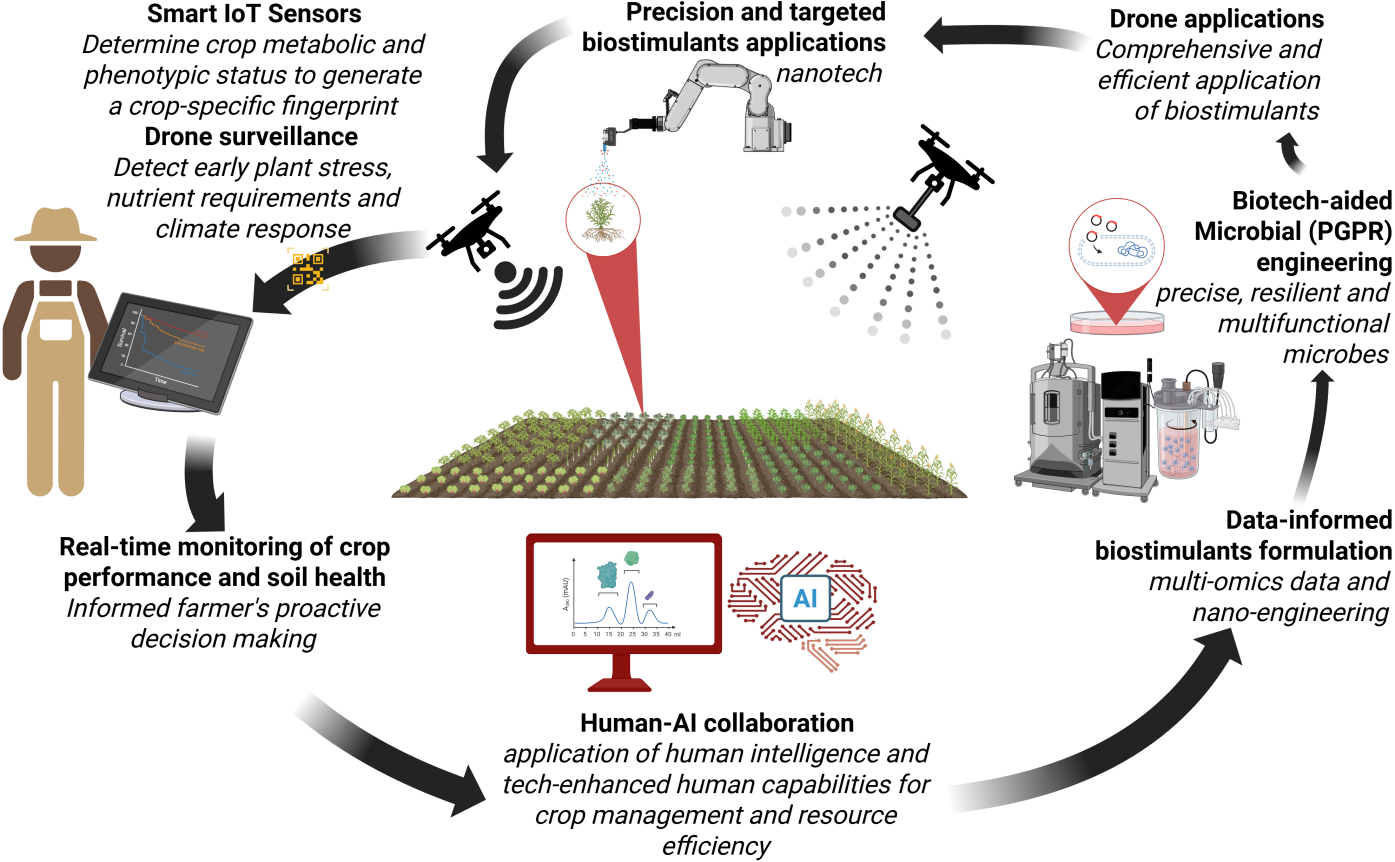
Integrated precision agriculture workflow combining IoT sensors, AI, and biotechnology for optimised crop management. Smart IoT sensors monitor crop metabolic and phenotypic status to generate crop-specific fingerprints, enabling drone surveillance for early detection of plant stress and nutrient requirements. This data informs precision biostimulant applications using nanotechnology and targeted delivery systems. Human-AI collaboration processes multi-omics data for informed decision-making, while biotech-aided microbial engineering (PGPR) develops resilient, multifunctional microbes. The system creates a continuous feedback loop for real-time crop performance monitoring, soil health assessment, and data-informed biostimulants formulation.

As evidenced by extensive literature and industry developments, conventional biostimulants have demonstrated significant efficacy in enhancing plant growth and stress resilience. Recent omics technologies are driving a transformative understanding of both microbial and non-microbial biostimulants, as well as their interactions with associated plants (Ruzzi et al., 2024). These insights are now steering the development of next-generation (Biostimulants 4.0) biostimulant formulations that are more targeted, efficient, predictive and aligned with the molecular needs of specific plant genotypes under diverse environmental conditions (de Andrade Silva et al., 2023; Mandal et al., 2023; Mashabela et al., 2023). On the horizon, Biostimulants 4.0 - first coined here - represents a new paradigm wherein bioformulations are increasingly informed by systems biology, multi-omics data, nanotechnology, and precision agriculture principles (**Figure 4**). These biostimulants, no longer limited to a “one-size-fits-all” mechanism of application, are designed using data-driven approaches that consider genotype-specific responses and environmental triggers, with greater stability and environmentally adaptive delivery systems (Rouphael and Colla, 2020; Bajpai et al., 2024; Garg et al., 2024).

Parallel to these biotechnological advances, agriculture is undergoing a digital transformation, moving from Agriculture 4.0 toward the emerging concept of Agriculture 5.0. The digital farming era is characterised by the Internet of Things (IoT), smart sensors, drone technology, AI-powered decision systems based on cloud computing and big data analytics, which facilitate climate-resilient practices (Wolfert et al., 2017). Agriculture 5.0 builds upon Agriculture 4.0, which focused on automation and technological innovation, by adopting a more inclusive approach that integrates human intelligence and sustainable practices (Rahmani et al., 2023), particularly concerned with global challenges such as climate change, resource depletion, and environmental degradation (Strazzullo et al., 2023; Rame et al., 2024). At its core, this approach leverages technology to enhance human capabilities while ensuring environmental protection and resource efficiency (**Figure 4**).

**Figure 5:**
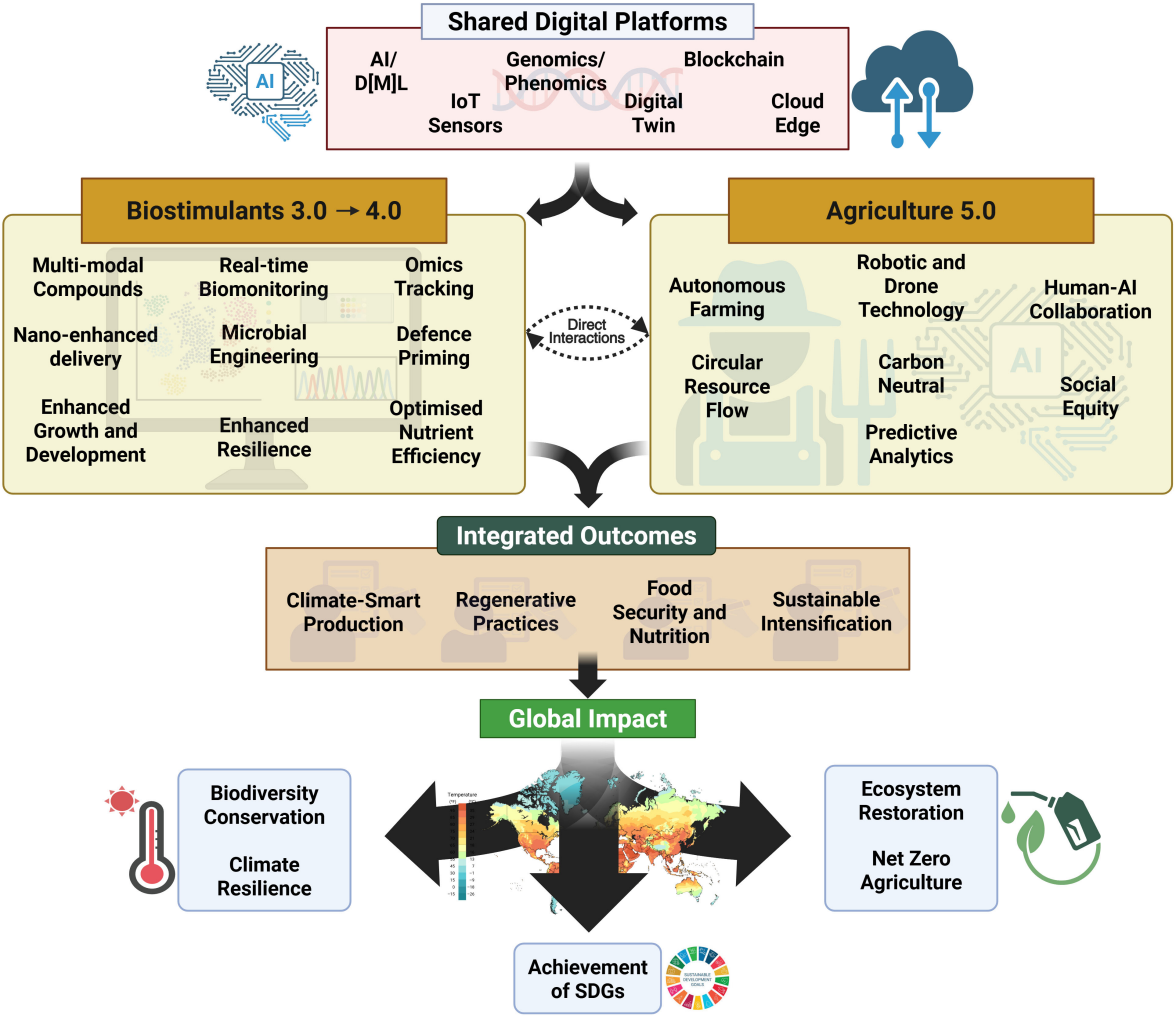
Conceptual framework for next-generation agricultural systems integrating biostimulants and digital technologies. The diagram illustrates how shared digital platforms (AI/ML, IoT sensors, genomics/phenomics, blockchain, digital twin, and cloud edge technologies) enable the convergence of Biostimulants 3.0-4.0 and Agriculture 5.0. Key innovations include multimodal compounds, real-time biomonitoring, nano-enhanced delivery, microbial engineering, and enhanced resilience for biostimulants. Meanwhile, advances in agriculture encompass autonomous farming, robotic/drone technology, human-AI collaboration, circular resource flow, carbon neutrality, and predictive analytics. This integration drives climate-smart production, regenerative practices, food security, and sustainable intensification, ultimately contributing to biodiversity conservation, climate resilience, ecosystem restoration, net-zero agriculture, and achievement of UN Sustainable Development Goals.

The prospects of Agriculture 5.0 are gaining interest from the science fraternity; the need for this trajectory is substantiated by the move from the 4^th^ to the 5^th^ industrial revolution (Industry 5.0) (Ramírez-Cedillon et al., 2025). However, there is relatively little literature on Agriculture 5.0 (Bissadu et al., 2025; Fountas et al., 2024; Haloui et al., 2024; Rame et al., 2024). As such, the role of biostimulants in this new age is yet to be fully explored or thoroughly defined. Nevertheless, the intersection of Biostimulants 3.0 and Agriculture 5.0 offers a transformative pathway for smart and sustainable crop management (**Figure 5**). Biostimulants, when integrated with digital farming platforms, enable real-time monitoring of plant responses, site-specific application based on predictive algorithms, and adaptive management protocols responsive to biotic or abiotic stressors (Strazzullo et al., 2023; Rame et al., 2024). This integrated framework holds significant promise in optimising inputs, enhancing plant resilience, and contributing to the United Nations Sustainable Development Goals (Ait-El-Mokhtar et al., 2024; Garrido et al., 2024).

Several real-world case studies now exemplify the potential of integrating biostimulants (3.0) with smart farming platforms. Projects like SmartFarm 2.0 in Europe combine PGPR-based biostimulants with drone-assisted growth monitoring and AI-powered phenological models to optimise application timing and nutrient efficiency. Likewise, Netafim’s fertigation systems enable the precise delivery of biostimulants based on real-time soil moisture and climate data, significantly enhancing efficacy while conserving resources. In North and South America, Taranis AI and AgriEdge use drone surveillance and big data analytics to detect early plant stress and optimise input use. Notably, Elicit Plant has developed a heat stress-responsive phytosterol biostimulant that is scheduled via AI-driven climate models, helping cereals maintain productivity under climate extremes. Despite these encouraging advances, their implementation in the Global South remains limited due to gaps in digital infrastructure, cost barriers, and a lack of policy support. This underscores the need for localised innovation, capacity building, and strategic public-private partnerships to enable equitable access to smart bio-based agricultural systems.

By enabling adaptive, data-informed plant management, Biostimulants 3.0, within the agriculture 5.0 framework, has the potential to usher in a new era of agroecological sustainability, productivity, and resilience. However, the promise of Agriculture 5.0 will remain aspirational unless regulatory and policy frameworks keep pace with technological innovation. Harmonised definitions, standardised testing protocols, and digital traceability systems are essential to ensure that advanced biostimulants transition from experimental trials to scalable, globally accessible solutions.

## 7 Conclusions and future research directions

This review addresses key critical gaps in the advancement and broad-scale adoption of biostimulants, including the absence of temporal-technological frameworks that connect biostimulants development with agricultural evolution, the insufficient mechanistic understanding linking molecular action to precision applications, and unclear integration strategies within digital agriculture. As such, our generational framework (Biostimulants 1.0-4.0) charts progression from empirical extracts through mechanistically validated formulations to emerging digitally integrated systems. This trajectory reflects a shift from observational benefits toward precision bioengineering and digitally integrated systems. The integration of biostimulants into climate-smart agriculture is particularly promising, aligning with its core pillars of increased productivity, enhanced resilience, and reduced environmental impact. By reducing reliance on chemical inputs and supporting crop performance under drought, salinity, and extreme temperatures, biostimulants directly contribute to sustainable intensification strategies and regenerative farming models.

Several interconnected mechanisms underpin the efficacy of biostimulants, including hormonal network modulation, nutrient efficiency, stress metabolite induction, rhizosphere microbiome engineering, and epigenetic priming, which confers transgenerational tolerance. These mechanisms can operate synergistically, enabling rational design for multi-functional biostimulant formulations. Future research in this regard encompasses key objectives, including systems-level technological integration, standardised protocols, safety assessments, AI-powered discovery of responsive nanocarriers, IoT-coupled predictive systems, genotype-specific formulations, programmable microbial circuits, climate-adaptive formulations, and bioeconomy integration. The realisation of this precision-aided biostimulants design can be accelerated by regulatory harmonisation, which requires joint expert committees on biostimulants (FAO/WHO) implementing risk frameworks. Equitable access demands technology transfer, regional hubs, and blended financing, incentivising adoption. These advances will directly support key SDGs such as SDG 2, 3, 13 and 15. However, realising this potential requires coordinated action: scientists prioritising mechanistic clarity through systems biology-focused analysis; industry’s investment into scalability, accessible formulations and policymakers advancing harmonisation. Integration with Agriculture 5.0 presents an unprecedented opportunity for climate-smart and climate-resilient agriculture and sustainable systems.

As climate pressures intensify, the roadmap outlined here provides a foundation for the future, though its implementation depends on sustained commitment, recognising biostimulants as integral components of reimagined agricultural systems. The future lies in thoughtful techno-ecology and the promise that the next generation of biostimulants embodies.
